# Diffusion of Intraperitoneal Chemotherapy in Women with Advanced Ovarian Cancer in Community Settings 2003–2008: The Effect of the NCI Clinical Recommendation

**DOI:** 10.3389/fonc.2014.00043

**Published:** 2014-03-10

**Authors:** Erin J. Aiello Bowles, Karen J. Wernli, Heidi J. Gray, Andy Bogart, Thomas Delate, Maureen O’Keeffe-Rosetti, Larissa Nekhlyudov, Elizabeth Trice Loggers

**Affiliations:** ^1^Group Health Research Institute, Group Health Cooperative, Seattle, WA, USA; ^2^Fred Hutchinson Cancer Research Center, Seattle, WA, USA; ^3^University of Washington, Seattle, WA, USA; ^4^Pharmacy Department, Kaiser Permanente Colorado, Aurora, CO, USA; ^5^Center for Health Research, Kaiser Permanente Northwest, Portland, OR, USA; ^6^Department of Population Medicine, Harvard Medical School, Boston, MA, USA; ^7^Department of Medicine, Harvard Vanguard Medical Associates, Boston, MA, USA

**Keywords:** ovarian cancer, chemotherapy, intraperitoneal, diffusion, age, stage

## Abstract

**Purpose:** A 2006 National Cancer Institute clinical announcement recommended the use of combined intravenous (IV) and intraperitoneal (IP) chemotherapy over IV chemotherapy alone for women with International Federation of Gynecology and Obstetrics (FIGO) stage 3 optimally debulked ovarian cancer due to significant survival benefit demonstrated in multiple randomized clinical trials. We examined uptake of IP chemotherapy in community practice before and after this recommendation.

**Methods:** We identified 288 women with FIGO stage 2 or greater incident ovarian cancer diagnosed from 2003 to 2008 at three integrated delivery systems in the US. Administrative health plan data were used to determine patient characteristics and receipt of IV and IP chemotherapy within 12 months of diagnosis. We compared characteristics of women receiving IV chemotherapy alone vs. IP chemotherapy (with or without IV chemotherapy) and assessed temporal trends in IP chemotherapy use.

**Results:** Overall 12.5% (*n* = 36) of women received IP chemotherapy during the study period. IP chemotherapy use was non-existent between 2003 and 2005. Use of IP chemotherapy occurred among 26.9% of women diagnosed in 2006 and plateaued at 20.4% of women diagnosed in 2008. IP recipients were younger (mean age 55.9 vs. 63.5 years, *p* = < 0.001) and more likely to have stage 3 ovarian cancer (77.8 vs. 50.4% *p* = 0.039) compared to their IV-only chemotherapy counterparts.

**Conclusion:** Use of IP chemotherapy for newly diagnosed advanced stage ovarian cancer patients was uncommon in this community setting. Future research should identify potential patient, physician, and system barriers and facilitators to using IP chemotherapy in this setting.

## Introduction

Approximately 22,280 new ovarian cancers were diagnosed in 2012, and an estimated 15,500 women died from the disease (1). While ovarian cancer is uncommon, long-term cure is poor with an overall 5-year survival rate of 43.7% (1). Prior to 2006, women with late-stage ovarian cancer were treated primarily with intravenous (IV) chemotherapy, which generally included a combination of platinum and taxane given every 3 weeks for six courses (2). However, this treatment has resulted in only a 26.9% 5-year survival rate when prognosis is grim (1). The development and testing of new, more effective treatments is currently our best hope of improving ovarian cancer survival.

On January 4, 2006, the National Cancer Institute (NCI) released a clinical announcement that recommended combined IV and intraperitoneal (IP) chemotherapy for women with FIGO stage 3 optimally debulked ovarian cancer (3). The announcement was based on national randomized trial data with 415 women with stage 3 ovarian cancer and <1 cm residual disease (GOG 172). GOG 172 showed a 16-month increase in overall survival for women receiving IP chemotherapy over IV chemotherapy alone (both groups received paclitaxel plus cisplatin) in women with <1 cm residual disease (4). The survival rate represented a marked improvement over previous trials (GOG 104 and 114), which were limited to cisplatin-only IP therapy and showed improvements in overall survival of 8 and 11 months, respectively (5, 6). IP chemotherapy delivers treatment directly into the abdominal cavity and targets the tumor site much more directly than IV chemotherapy, which is delivered through the bloodstream. While IP chemotherapy promises a significant advancement in survival, the side effects are generally more significant than for IV chemotherapy. Clinical studies have shown that side effects for IP chemotherapy include infection, abdominal pain, and bowel damage, all of which may limit patient tolerability (4, 7). This has limited uptake of IP chemotherapy, even in academic settings, and created controversy about the generalizability of trial results, particularly in community settings (i.e., outside of cancer centers) where familiarity with ovarian cancer and intra-abdominal catheters is more limited.

The purpose of our study was to examine patterns of IP and IV chemotherapy treatment in three sites of the Cancer Research Network (CRN), a consortium of research groups associated with integrated delivery systems in the United States with over 11 million enrollees (8). We examined treatment patterns by patient characteristics and over time to better understand the use of IP chemotherapy in community practice for women with advanced ovarian cancer.

## Materials and Methods

The HMO CRN consists of the research programs, enrollee populations, and databases of 14 members of the HMO Research Network (8). An overall goal of the CRN is to conduct collaborative research to determine the effectiveness of preventive, curative, and supportive interventions for major cancers that span the natural history of those cancers among diverse populations and health systems. The 14 health plans, with nearly 11 million enrollees are distinguished by their longstanding commitment to prevention and research, and collaboration among themselves and with affiliated academic institutions. This study was conducted in three sites from the CRN: Group Health (Washington State), Kaiser Permanente Colorado, and Kaiser Permanente Northwest (Oregon/Southwest Washington). Institutional Review Board oversight and approval was ceded to Group Health for all sites involved in this study.

### Study population

We identified all women with incident ovarian cancer diagnosed between January 1, 2003 and December 31, 2008 from each site’s local Surveillance Epidemiology and End Results (SEER) or tumor registry (*N* = 921). We included women with epithelial and other morphologies of ovarian cancer who had records of IV chemotherapy and/or IP chemotherapy following diagnosis (*N* = 381). We excluded morphologies for myomatous neoplasms (ICD-O-3 code 8890), malignant lymphoma (ICD-O-3 9590 and 9680), sex cord stromal (ICD-O-3 8590, 8600, 8620, 8621, 8630, 8631, 8640, 8670, 8810), germ cell (ICD-O-3 8240, 8246, 9060, 9064, 9070, 9071, 9072, 9080, 9081, 9084, 9085, 9090, 9100), and follicular and marginal lymphoma (ICD-O-3 9690) (*N* = 4). We further excluded women with FIGO stage I disease (*N* = 52) and women who started chemotherapy treatment more than 12 months after their cancer diagnosis (*N* = 37). Even though IP chemotherapy is only recommended for stage 3 ovarian cancers, we included stages 2 and 4 to evaluate whether treatment was administered outside of guidelines. Most women were treated by oncologists employed by their health plan (most of whom are medical oncologists, not gynecologic oncologists). However, women may have received oncology care outside of the health plan (such as at a cancer center) based on their insurance coverage and preferences; treatment received outside of the health plans was collected for this analysis via claims. Our final sample size for analysis was 288 women.

### Data collection

We obtained data from each site’s virtual data warehouse (VDW), which has been described in detail elsewhere (9). Briefly, the VDW includes standardized variables derived from administrative databases at each CRN site. A programmer at Group Health wrote standardized code to execute at the other sites; programmers then transferred limited datasets to Group Health for analysis. Using VDW data, we linked tumor registry data to health plan data on demographics and enrollment to identify patient characteristics, including age at diagnosis, race, and ethnicity. We used tumor registry data to identify ovarian cancer stage at diagnosis and receipt of surgery. We linked to health plan utilization data to identify women’s encounters with the health plan within 1 year following diagnosis including ambulatory visits, emergency department visits, hospital stays, e-mails with providers, and telephone visits.

We collected data on chemotherapy administration (including claims) using validated VDW procedure codes (10–12). Chemotherapy procedure data included healthcare common procedure coding system (HCPCS) and current procedural terminology (CPT-4) codes. We used CPT-4 code 96445 to identify the receipt of IP chemotherapy. We explored whether CPT-4 codes 49419 and 49422 (insertion and removal of an IP catheter) indicated use of IP chemotherapy and decided not to include these codes because they were not used consistently among women who received IP chemotherapy (based on CPT-4 code 96445). We used CPT-4 codes 96408-96417 and HCPCS codes C8953–C8955, G0359–G0361, Q0084, Q0085, S9330, and S9331 to identify receipt of IV chemotherapy. For both IP and IV chemotherapy, we collected information on the date of first administration and total number of administrations.

### Statistical analysis

We compared the distribution of patient characteristics, tumor characteristics, and healthcare utilization between women who received any IP chemotherapy (with or without IV chemotherapy) and women who received IV chemotherapy alone. We used the two-sample Wilcoxon rank-sum test to evaluate differences in continuous variables (e.g., age) and Fisher’s exact test to evaluate differences in categorical variables (e.g., stage). We calculated the cumulative count of first IP and IV procedures separately over time. If a woman had more than one IP or IV procedure, only the first one of each was counted. All analyses were descriptive and unadjusted. All statistical analyses were conducted in SAS software version 9.3 (SAS Institute, Cary, NC, USA).

## Results

A total of 288 women were identified with FIGO stage 2 or greater incident ovarian cancer between 2003 and 2008. Of these, 36 (12.5%) women received IP chemotherapy with or without IV chemotherapy and 254 (87.5%) received IV chemotherapy only (Table 1). Women who received IP chemotherapy were younger (mean age 55.9 years) than women who received IV chemotherapy alone (mean age 63.5 years, *p* < 0.001). Women who received IP chemotherapy differed in disease stage compared with those who received IV chemotherapy alone (*p* = 0.039); a larger percentage of women receiving IP had stage 3 disease (77.8%) than women receiving IV chemotherapy alone (50.4%).

**Table 1:** **Patient characteristics by IP chemotherapy status**.

	All	IV chemotherapy without IP	IP with or without IV chemotherapy	*p*-Value comparing IV alone to IP
	*N* = 288	*N* = 252	*N* = 36	
Age at diagnosis, mean (SD)	62.7 (11.6)	63.7 (11.7)	55.9 (8.2)	<0.0001
	***N* (Column %)**	***N* (Column %)**	***N* (Column %)**	
Age group at diagnosis, *n* (%)				0.001
under 40	3 (1.0)	3 (1.2)	0	
40–9	35 (12.2)	26 (10.3)	9 (25)	
50–59	81 (28.1)	67 (26.6)	14 (38.9)	
60–69	85 (29.5)	73 (29.0)	12 (33.3)	
70–79	61 (21.2)	60 (23.8)	1 (2.8)	
80 And higher	23 (8.0)	23 (9.1)	0	
White, *n* (%)	240 (83.3)	207 (82.1)	33 (91.7)	0.48
FIGO/AJCC stage at diagnosis, *n* (%)				0.039
2	24 (8.3)	21 (8.3)	3 (8.3)	
3	155 (53.8)	127 (50.4)	28 (77.8)	
4	69 (24.0)	65 (25.8)	4 (11.1)	
Unknown	40 (13.9)	39 (15.5)	1 (2.8)	
Surgery receipt, *n* (%)	250 (86.8)	214 (84.9)	36 (100.0)	0.007
Days from diagnosis to first chemotherapy, mean (SD)	59.0 (56.3)	59.3 (57.2)	56.2 (47.9)	0.75
Days from diagnosis to first chemotherapy, *n* (%)				0.42
0–30 days	62 (21.5)	57 (22.6)	5 (13.9)	
31–60 days	127 (44.1)	111 (44.0)	16 (44.4)	
61–90 days	32 (11.1)	31 (12.3)	1 (2.8)	
91–180 days	21 (7.3)	20 (7.9)	1 (2.8)	
181–365 days	13 (4.5)	11 (4.4)	2 (5.6)	
Unknown	33 (11.5)	22 (8.7)	11 (30.6)	
IV chemo administrations, *n* (%)				<0.0001
None	4 (1.4)	0	4 (11.1)	
1–5	47 (16.3)	42 (16.7)	5 (13.9)	
6–10	40 (13.9)	30 (11.9)	10 (27.8)	
11 Or more	197 (68.4)	180 (71.4)	17 (47.2)	
IP Chemo administrations, *n* (%)				<0.0001
None	252 (87.5)	252 (100.0)	0	
1–5	20 (6.9)	0	20 (55.6)	
6–10	11 (3.8)	0	11 (30.6)	
11 Or more	5 (1.7)	0	5 (13.9)	
Encounters within 1 year following diagnosis, mean [minimum, median, maximum]				
Ambulatory visits	30.6 [4,27,111]	29.5 [4, 26,90]	38.0 [12, 36, 111]	0.003
Emergency department	0.9 [0,0,17]	0.9 [0,0,17]	1.0 [0, 0, 4]	0.60
Email encounters	0.5 [0,0,36]	0.2 [0,0,15]	2.4 [0, 0, 36]	0.0003
Acute inpatient stay	1.7 [0,1,8]	1.7 [0,1,8]	2.1 [1, 2, 5]	0.018
Telephone encounters	12.0 [0,6,97]	11.4 [0,6,97]	16.1 [0, 9, 75]	0.38
	***N***	***N* (row %)**	***N* (row %)**	
Study site, *n* (%)				<0.0001
A	138	121 (87.7)	17 (12.3)	
B	92	88 (95.7)	4 (4.3)	
C	58	43 (74.1)	15 (25.9)	
Year of diagnosis, *n* (%)				<0.0001
2003	39	39 (100.0)	0	
2004	42	42 (100.0)	0	
2005	49	48 (98.0)	1 (2.0)	
2006	51	37 (72.6)	14 (27.4)	
2007	53	43 (81.1)	10 (18.9)	
2008	54	43 (79.6)	11 (20.4)	

*IP, intraperitoneal; IV, intravenous; SD, standard deviation; FIGO, International Federation of Gynecology and Obstetrics; AJCC, American Joint Committee on Cancer*.

**P*-values for categorical items are from Fisher’s exact test, and those for continuous items are from the Wilcoxon rank-sum test. Frequencies from “unknown” categories do not contribute to statistical tests*.

Treatment patterns differed somewhat by group (Table 1). All women who received IP chemotherapy received surgery as their primary therapy; 15.1% of women who received IV chemotherapy did not have surgery. The time to chemotherapy initiation was similar between groups (mean 56.2 days for IP chemotherapy and 59.1 days for IV chemotherapy).

**Figure 1:**
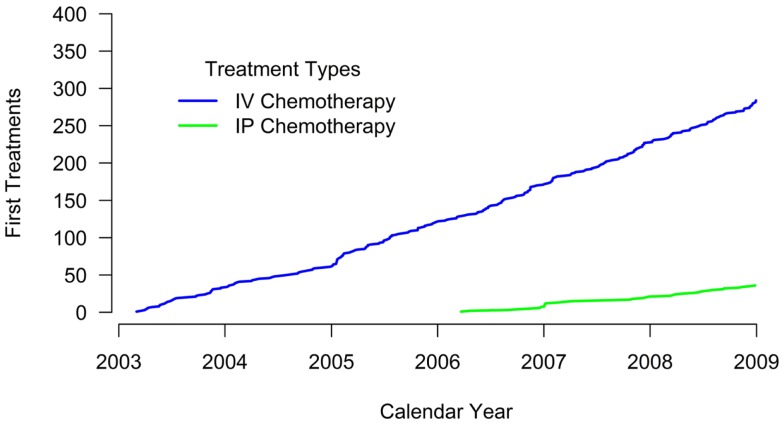
**Cumulative count of first treatments**. The blue line shows the cumulative count of initial IV chemotherapy administrations over time. The green line shows the cumulative count of initial IP chemotherapy administrations over time. Treatment receipt was not mutually exclusive. For example, if a woman received both IV and IP chemotherapy, her first treatment for each modality is included in the figure.

No evidence of IP chemotherapy use was found from 2003 through 2005. Use was first identified in 2006 when IP chemotherapy was received by 27.4% of women in our sample. In 2007, 18.9% of women received IP chemotherapy while 20.4% of women diagnosed in 2008 received IP chemotherapy. Figure 1 illustrates that the use of IP chemotherapy was much less common than IV chemotherapy over time. Treatment differed by study site and year of diagnosis. Among women at each site, 12.3% received IP chemotherapy at site A, 4.3% at site B, and 25.9% at site C.

## Discussion

In this study, we examined the use of IP chemotherapy for advanced stage ovarian cancer in a community setting. Our results suggest that despite the 2006 NCI announcement recommending IP chemotherapy as the new standard of care treatment for women with stage 3 optimally debulked tumors, use of IP chemotherapy was uncommon in women insured at three CRN healthcare delivery systems immediately following the announcement. Of the estimated 22,000 new diagnoses in the U.S. in 2012, approximately 60% were likely diagnosed with distant disease and could have been eligible for IP chemotherapy treatment (1). In our community-based patient population, use of IP chemotherapy increased after the clinical guideline recommendation, but plateaued at about 20% of women, with notable differences in receipt of IP chemotherapy by age.

The reasons for differences we observed in IP chemotherapy treatment by age may be related to aggressiveness of care. Younger women may be more willing to tolerate the additional toxicities of IP chemotherapy than older women and their providers may be more likely to recommend this aggressive treatment because of their younger age. Interestingly, while most women who received IP chemotherapy had stage 3 disease, about 20% of women who received IP chemotherapy were stages 2 or 4. This finding suggests that some treatment was received outside of guidelines (13) or that stage was incorrectly or incompletely documented when IP chemotherapy treatment was initiated.

The reasons for low IP chemotherapy use in these community settings are not clear. Providers may be reluctant to offer IP chemotherapy given the potential for toxicity and complexities in administration. Centers may not be adequately trained in IP chemotherapy administration performed in an inpatient setting. The low use is concerning as it suggests women with advanced ovarian cancer potentially eligible for this treatment may not have access to appropriate care or may not be offered IP chemotherapy, for a number of physician or system-related reasons. Alternatively, after additional counseling on and follow-up for side effects, women may refuse IP chemotherapy.

Our study does have limitations. Most notably, we did not collect data on patient, provider or systemic reasons for not using IP chemotherapy. We do not have comprehensive information on the eligibility of these women for IP chemotherapy (including information on residual disease following surgery) or whether they had local access to properly trained surgeons with experience in catheter placement and care or IP chemotherapy administration. We did not collect information on treating physicians and have no way of identifying the small number of patients treated outside of their health plan through the treatment codes we used. The small number of IP users limits our ability to conduct sub- and multi-variable analyses. Between 20 and 40% of women presenting with advanced ovarian cancer will have suboptimal surgical debulking, which would make them ineligible for IP treatment (13). Unfortunately, we were not able to determine this important surgical variable in this study. Finally, we do not have data beyond 2008; thus these results are only generalizable to the period of time immediately following the clinical announcement. Use of IP chemotherapy may have increased since then, and may continue to do so in the future; however, we are unable to examine this in our study.

Despite these limitations, our study has several strengths. To our knowledge, this was the first study to document the diffusion of IP chemotherapy in community-based clinical settings. We were able to conduct this analysis because of our data linkages between procedure data, tumor registry data, and other administrative health plan data using the CRN VDW, a validated source of information regarding community cancer patients. We also had complete and valid data on chemotherapy use from CPT and HCPCS codes, which have been validated in prior studies (10–12) and include any claims for treatment from external treatment centers (such as a cancer center). In addition, our study is a multi-site study, improving generalizability of our results. Finally while other diffusion studies may be influenced by provider reimbursement for treatment, our three study sites employed salaried physicians during the study period and should not have been influenced by reimbursement.

In conclusion, our study shows treatment with IP chemotherapy for women with advanced stage ovarian cancer is uncommon in our three community-based integrated delivery systems. This may be due to system, provider, and/or patient factors. However, the lack of uptake IP chemotherapy use is concerning as ovarian cancer patients may not be receiving therapy that would give them the best chance at survival. Additional research into the barriers and facilitators to IP chemotherapy treatment is needed.

## Author Contributions

The authors contributed to this manuscript as follows: conception and design of the work (Erin J. Aiello Bowles, Karen J. Wernli, Larissa Nekhlyudov, Elizabeth Trice Loggers); acquisition, analysis, or interpretation of data for the work (Erin J. Aiello Bowles, Karen J. Wernli, Heidi J. Gray, Andy Bogart, Thomas Delate, Maureen O’Keeffe-Rosetti, Larissa Nekhlyudov, Elizabeth Trice Loggers); drafting the work or revising it critically for important intellectual content (Erin J. Aiello Bowles, Karen J. Wernli, Heidi J. Gray, Andy Bogart, Thomas Delate, Maureen O’Keeffe-Rosetti, Larissa Nekhlyudov, Elizabeth Trice Loggers); final approval of the version to be published (Erin J. Aiello Bowles, Karen J. Wernli, Heidi J. Gray, Andy Bogart, Thomas Delate, Maureen O’Keeffe-Rosetti, Larissa Nekhlyudov, Elizabeth Trice Loggers).

## Conflict of Interest Statement

The authors declare that the research was conducted in the absence of any commercial or financial relationships that could be construed as a potential conflict of interest.
